# Three-dimensional non-parametric method for limbus detection

**DOI:** 10.1371/journal.pone.0207710

**Published:** 2018-11-26

**Authors:** Ahmed Abass, Bernardo T. Lopes, Ashkan Eliasy, Richard Wu, Steve Jones, John Clamp, Renato Ambrósio, Ahmed Elsheikh

**Affiliations:** 1 School of Engineering, University of Liverpool, Liverpool, United Kingdom; 2 Federal University of São Paulo, São Paulo, Brazil; 3 Central Taiwan University of Science and Technology, Taichung, Taiwan; 4 Pacific University, College of Optometry, Forest Grove, Oregon, United States of America; 5 UltraVision CLPL, Leighton Buzzard, United Kingdom; 6 National Institute for Health Research (NIHR) Biomedical Research Centre at Moorfields Eye Hospital NHS foundation Trust and UCL Institute of Ophthalmology, London, United Kingdom; Cedars-Sinai Medical Center, UNITED STATES

## Abstract

**Purpose:**

To present a novel non-parametric algorithm for detecting the position of the human eye limbus in three dimensions and a new dynamic method for measuring the full 360° visible iris boundary known as white-to-white distance along the eye horizontal line.

**Methods:**

The study included 88 participants aged 23 to 65 years (37.7±9.7), 47 females and 41 males. Clinical characteristics, height data and the apex coordinates and 1024×1280 pixel digital images of the eyes were taken by an Eye Surface Profiler and processed by custom-built MATLAB codes. A dynamic light intensity frequency based white-to-white detection process and a novel three-dimensional method for limbus detection is presented.

**Results:**

Evidence of significant differences (p<0.001) between nasal-temporal and superior-inferior white-to-white distances in both right and left eyes were found (nasal-temporal direction; 11.74±0.42 mm in right eyes and 11.82±0.47 mm in left eyes & superior-inferior direction; 11.52±0.45 mm in right eyes and 11.55±0.46 mm in left eyes). Average limbus nasal-temporal diameters were 13.64±0.55 mm for right eyes, and 13.74±0.40 mm for left eyes, however the superior-inferior diameters were 13.65±0.54 mm, 13.75±0.38 mm for right and left eyes, respectively. No significant difference in limbus contours has been observed either between the nasal-temporal direction (p = 0.91) and the superior-inferior direction (p = 0.83) or between the right (p = 0.18) and left eyes (p = 0.16). Evidence of tilt towards the nasal-temporal side in the three-dimensional shape of the limbus was found. The right eyes mean limbus contour tilt around the X-axis was -0.3±1.35° however, their mean limbus contour tilt around the Y-axis was 1.76±0.9°. Likewise, the left eyes mean limbus contour tilt around the X-axis was 0.77±1.25° and the mean limbus contour tilt around the Y-axis was -1.54±0.89°.

**Conclusions:**

The white-to-white distance in the human eye is significantly larger in the nasal-temporal direction than in the superior-inferior direction. The human limbus diameter was found not to vary significantly in these directions. The 3D measures show that the limbus contour does not lay in one plane and tends to be higher on the nasal-inferior side of the eye.

## Introduction

The human eyeball casing consists of two connected components; the cornea and the sclera. The cornea is the most powerful refractive element of the eye as it provides more than 70% of the eye’s refractive power [1, 2], while the sclera contributes to the ocular mechanical strength which endures the intra-ocular pressure [3]. Furthermore, the sclera efficiently handles the forces applied by the extraocular muscles during eye movement without distorting the corneal surface. Although the limbus is commonly known as the edge of the cornea where it joins the sclera, it may be defined in different ways according to its identification method. From a microscopic approach, it is the junction between the cornea and the sclera [4], but in terms of transparency, it is a transition border between the transparent cornea and the opaque sclera [5]. With regards to the eye surface profile, the limbus is defined as a smooth transition zone with a more obtuse curvature from the cornea to the sclera [6]. Because it is relatively close to the transparent zone of the cornea, the limbus border is frequently approximated to the visible iris boundary. However, the iris lies in a different plane a few millimetres away from the limbus plane with no direct contact between them apart from the connection through the ciliary muscles.

Consejo et al [7] listed a comprehensive survey of vertical and horizontal visible iris diameter values, while the white-to-white corneal distances were assessed in previous studies. In all of these clinical and computer-based methods [7–18], white-to-white corneal distances were estimated based on the imaging light intensity transition from the dim iris to the bright sclera. However, many different forms of digital imaging analysis have been used extensively for detecting the limbus of human eyes [5, 19–24]; all of these methods detect the visible iris diameter not the limbus profile. Moreover, the grey area (on an eye image) between the dark iris and the white sclera forces analysts to choose the boundary position subjectively. Positions of these thresholds between dark and white pixels directly affect the results of the limbus detection method. Most of the available methods of estimating the limbus shape were limited to the assumption of representing the limbus as a two-dimensional profile. In 2002, Morelande and Iskander presented a method that used the image of the eye and repetitive ellipse fitting for detecting the limbus [25]. Then, Jesus and Iskander provided a parametric algorithm for estimating the limbus shape. They used Zernike polynomials to represent the corneal and the scleral boundaries of the anterior eye in circular and elliptical fields independently [6]. They concluded that the circular model provided a more robust estimation of the limbus position. Recently, an asymmetric mean shape of the human limbus was introduced by Consejo who fitted the human limbus shape to a second-order Fourier series [26]. These methods only estimated the two-dimensional limbus shape and most of them approximated it to the best-fitted circular shape.

Eye profile height data were used to detect the limbus by Consejo [27]. The methods used were based on calculating the cumulative root mean square of the residual error between a Zernike polynomial fitted surface and the original polynomial fit of the eye surface resulting in high fitting errors around the limbus. As the method has not been tested on irregular corneas, it was not clear if this fitting-error algorithm could be used efficiently in such cases. Recently, Consejo concluded that second order Fourier series was the most accurate model to describe the shape of the human limbus [26], however, the study was limited to left eyes only, has no mechanism to deal with eye profile data affected by eyelids and has not been compared to any non-parametric methods. Moreover, the vast majority of limbus detection studies do not acknowledge the limbus width and its inclusion as a part of the corneal span, hence, underestimating the limbus diameter [28].

Even the clinical and computer-based methods described to detect the visible iris present issues [7–18]. They considered the imaging light intensity transition from the dim iris to the bright sclera, but in order to deal with the grey area that exists between them on an eye image the analysts have to choose a subjective threshold. This leads to inaccuracies in the identification of the true visible iris diameter, in addition to the important fact that the measurement of the visible iris diameter is not a measurement of the limbus profile [5, 19–24].

Some researchers proposed different mathematical approaches to identify the limbus based on repetitive ellipses, Zernike polynomials and second-order Fourier series fitting [26]. However, these methods only estimate the two-dimensional limbus shape and most of them approximate it to the best-fitted circular shape.

This study presents a novel non-parametric algorithm for detecting the human eye limbus in three dimensions (3D) based exclusively on eye profile data as taken by the Eye Surface Profiler (Eaglet Eye BV, AP Houten, The Netherlands). The study also presents a dynamic method for measuring the visible iris boundary (white-to-white) corneal distances from calibrated digital images of the eye. Then the differences between the topographic limbus-to-limbus profile and the white-to-white corneal boundary are determined in all directions.

## Materials and methods

### Participants

This record review study was conducted according to the tenets of the Declaration of Helsinki and was approved by the IRB (Institutional Review Board) and Human Ethics Committee of the Federal University of São Paulo (UNIFESP, SP, Brazil). The study included 88 participants aged 23 to 65 years (37.7 mean ±9.7 STD), 47 females and 41 males. Participants suffering from ocular diseases or having a history of trauma or ocular surgery were excluded. The data were collected and anonymised at Brigthen Optix Corporation in Taipei, Taiwan where participants were told not to wear contact lens for two weeks before the profile measurement, and those who were wearing rigid gas-permeable (RGP) contact lens were asked not to wear them for four weeks before the scan.

Eye profiles were taken in a darkened room as any light falling on the profiler may affect the measurement. As the profiler being used must be attached to a computer, the computer monitor was positioned to direct its light away of the participant’s face and set to a low brightness level. The clinician made sure that the subject was in the correct position for measuring before applying any drops to avoid wasting time after applying them. Each participant was asked to set their head on the chinrest and headrest before their level was adjusted manually. Each participant saw a red-cross target in the instrument screen while the clinician saw it on the computer monitor. The precise alignment with the fixation axis was achieved clinically by making sure that the red-cross target was lined up with the centre of two white orientation dots resulting from focus lights shone by the instrument (illumination spots). This made sure that the fixation first Purkinje images were aligned vertically on top of the each other by aligning the profiler’s two fixation spots straight up. At that moment, the subject was asked to sit back before applying one unpreserved lubricating drop to their lower fornix (Lubristil, 1 mg/mL sodium hyaluronate) as the eye scan process using the Eye Surface Profiler (Eaglet Eye BV, AP Houten, The Netherlands) requires the instillation of fluorescein with a viscous solution. The subject was asked to look up then the clinician gently dabbed fluorescein, using ophthalmic strips (Bioglo, HUB Pharmaceuticals, Inc.), on the eye three times in the lower fornix and then the participant was asked to look down and dabbed three times in the upper fornix. By asking the subject to blink twice, the clinician ensured a good coverage of fluorescein over the eye anterior surface. While three measurements were being taken for each of their eyes, participants were instructed to open their eyelids wide to ensure surface data coverage up to a few millimetres beyond the limbal zone.

### Data collection

The data were exported from the ESP software in MATLAB binary data container format (*.mat) where the geometrical characteristics of eyes, as measured by the ESP system, were stored, see Table 1. In addition to the height data and the apex coordinates, a 1024×1280 pixel digital image of the eye and its horizontal and vertical calibration factors were extracted. The data has been processed by custom built MATLAB codes. The parameters that were extracted from the ESP software were only used for reporting the clinical parameters as shown in Table 1, and were not used for obtaining any result presented in this study.

**Table 1:** Geometrical characteristics of eyes as measured by the ESP system.

Geometrical characteristic	Right eyes	Left eyes
	Mean ± STD	Mean ± STD
	Min : Max	Min : Max
Horizontal visible iris diameter HVID (mm)	11.99 ± 0.40	11.97 ± 0.41
	10.80 : 12.63	10.62 : 12.63
Astigmatism (Dioptre)	-1.72 ± 0.71	-1.82 ± 0.69
	-3.97 : -0.13	-4.27 : -0.30
Axis (°)	96.37 ± 13.95	88.79 ± 6.85
	80.99 : 171.42	78.72 : 125.92
Sphere (Dioptre)	43.08 ± 1.66	43.12 ± 1.77
	38.48 : 48.33	37.91 : 48.48
Sim-K astigmatism (Dioptre)	-2.68 ± 1.07	-2.95 ± 1.03
	-6.02 : -0.42	-5.59 : -0.55
Sim-K angle (°)	93.45 ± 15.54	91.03 ± 7.00
	70.71 : 178.14	75.76 : 114.26
Sim-K flat radius (mm)	8.41 ± 0.40	8.44 ± 0.40
	7.41 : 9.60	7.49 : 9.67
Sim-K steep radius (mm)	7.88 ± 0.35	7.86 ± 0.37
	7.01 : 9.01	7.10 : 9.02

### White-to-white detection

**Fig 1:**
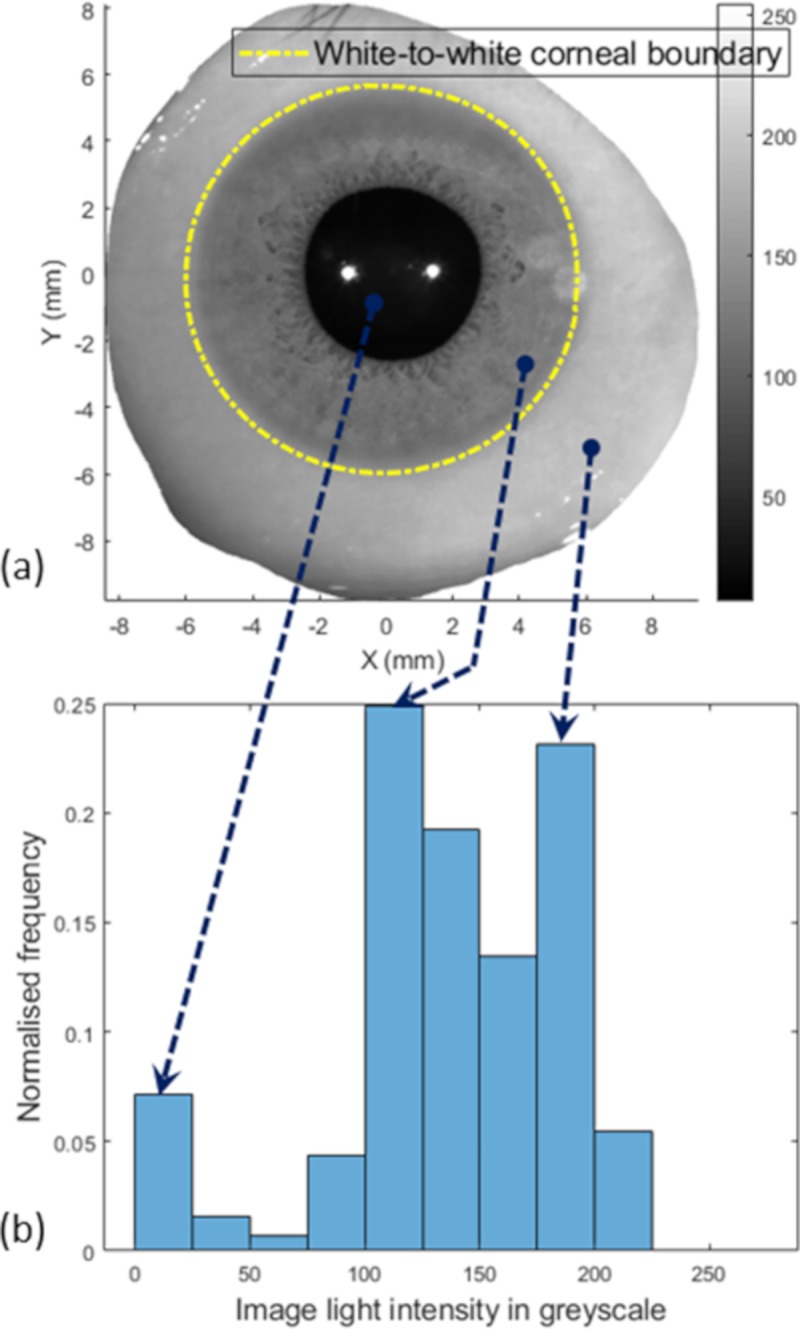
(a) An eye image taken by the ESP digital camera; (b) Frequency of different light intensity values where the first peak corresponds to pupil’s colour, the second peak corresponds to the iris’s colour and the last peak corresponds to the sclera’s colour.

As the image provided by the Eye Surface Profiler was often in greyscale, the original iris colour did not appear in the eye image and the equivalent greyscale colour appeared instead. Values of each pixel, the smallest elements of an image, are varying according to the light intensity in a grey scaled photograph between 255 for white and 0 for black, and each part of the eye appeared in a different greyscale range according to its colour. To distinguish between two parts by their colour, a threshold needs to be set to a certain value between the colour values of these two parts. Using a fixed value for the threshold would not take into account the variation of the level of the light intensity in the room during the eye profile scan, the participant’s iris colour and the settings of the camera. Therefore in this study, a dynamic thresholding algorithm was used by considering the frequency of the light intensity for each image and identifying the main peaks and relate them to their areas, see Fig 1. Looking at the key components of an eye image, the pupil, the iris and the sclera, the pupil is often the darkest area of the image, therefore, its colour frequency is usually the lower frequency in the image spectrum. The next two frequency peaks represent the iris and sclera, respectively. Whatever the iris colour and the lighting conditions during the eye profile measurement process, the relative order of these three frequency peaks stays the same. White-to-white contour profile was determined by finding the light intensity at the middle frequency peak then detecting the boundary of the area that contain this light intensity. The boundary line of this middle frequency area represented the border between the white sclera and the dark iris.

### Limbus detection

**Fig 2:**
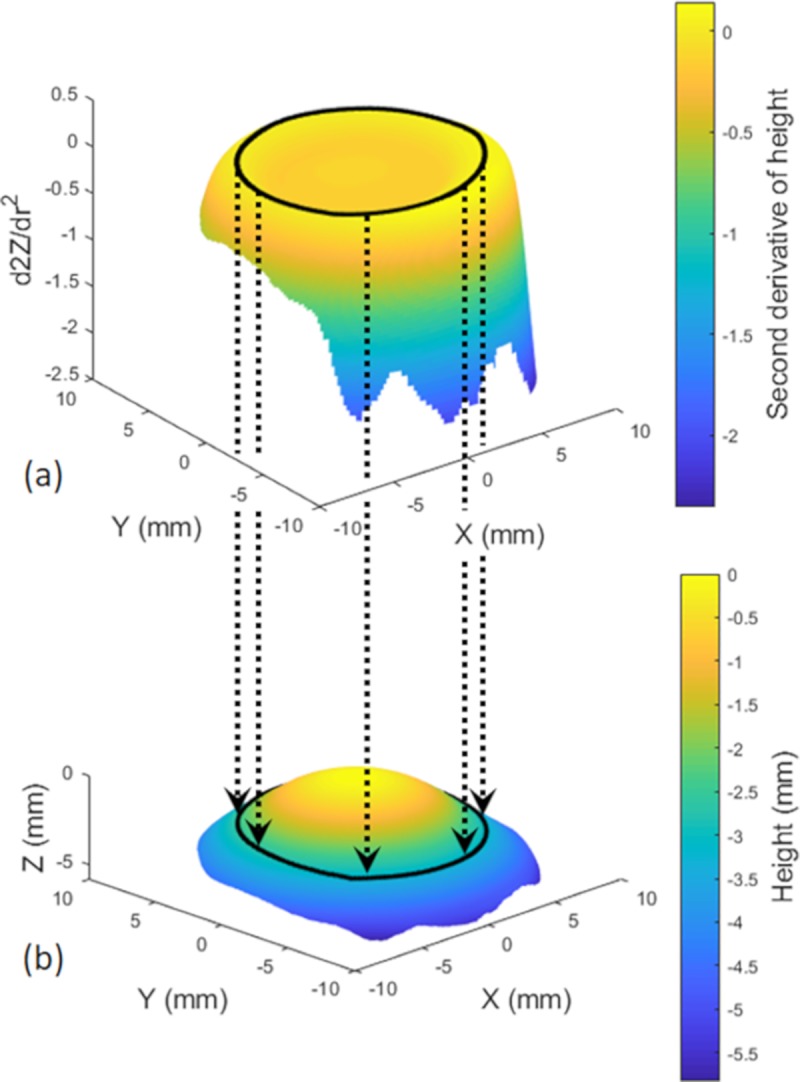
Detected limbus (black contour). (a) Second derivative of the anterior eye surface height data; (b) Anterior eye surface constructed by height data as measured by the Eye Surface Profiler.

**Fig 3:**
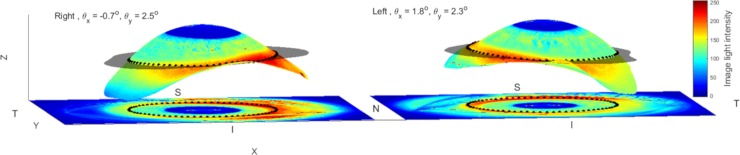
Both eyes of a 37 years old female participant. Location of the mean 3D limbus contour (black dots) fitted to 3D plane (transparent black) for right and left eyes. N, T, S, I stand for nasal, temporal, superior and inferior sides respectively. For displaying purposes, the digital image of the eye is projected onto the eye surface and the 3D limbus contour is projected onto the eye image.

The Eye Surface Profiler is able to collect data for the cornea and a portion of the sclera, Fig 2A, which allows the limbus to be detected through the ESP’s height data (*h*). The presented limbus detection algorithm is based on the cornea and the sclera having different curvatures and the limbus boundary is the point where the corneal curvature changes to the scleral curvature. Knowing that the eye surface tangent gradient $\frac{dh}{dr}$ (1^st^ derivative of the height *h* in respect to the corneal polar radius *r*) is changing from zero at the apex to a local maximum just before the limbus. It decreases gradually at the limbus then increases again as it moves onto the sclera. As the limbus is the place where the rate of change of the 1^st^ derivative with respect of the corneal radius $\frac{dh}{dr}$ is a minimum, the limbus can be detected by locating the turning point of the height 2^nd^ derivative $\frac{d^{2}h}{dr^{2}}$ at each meridian, Fig 2B. Thus, all detected limbus points on all meridians for each eye forms the limbus contour, this contour was fitted to a plane then tilt angles of this plane were determined. Limbus contour tilt angles θ_*x*_,θ_*y*_ around the X-axis and the Y-axis were determined starting from the positive Y-axis and X-axis correspondingly in a counter-clockwise manner, Fig 3.

### Statistical analysis

Statistical analysis was performed using MATLAB Statistics and Machine Learning Toolbox (MathWorks, Natick, USA). The null hypothesis probability (p) at a 95% confidence level was calculated. The two-sample t-test was used in order to investigate the significance between pairs of data sets to check whether the results represent independent records. The probability p is an element of the period [0,1] where values of p higher than 0.05 indicates the validity of the null hypothesis [29].

## Results

The results from this study showed that the average nasal-temporal white-to-white distances among participants were 11.74±0.42 mm for right eyes and 11.82±0.47 mm for left eyes, however the superior-inferior white-to-white distance were 11.52±0.45 mm and 11.55±0.46 mm for right and left eyes, respectively. So, there was a significant difference between the nasal-temporal and superior-inferior directions for both right and left eyes (p<0.001).

Limbus average distances in nasal-temporal direction were 13.64±0.55 mm for right eyes, and 13.74±0.40 mm for left eyes, however the superior-inferior spans were 13.65±0.54 mm, 13.75±0.38 mm for right and left eyes, respectively. No significant difference has been observed in the limbus contours either between the nasal-temporal direction (p = 0.91) and superior-inferior direction (p = 0.83) or between right (p = 0.18) and left eyes (p = 0.16), Table 2.

**Table 2:** Limbus to limbus edges, white-to-white edges and 3D limbus sagittal depth.

	Limbus-to-limbus edges			White-to-white edges			Limbus-to-limbus–White-to-white			3D Limbus sagittal depth			
Orientation	Right eyes (mm)	Left eyes (mm)	p	Right eyes (mm)	Left eyes (mm)	p	Right eyes (mm)	Left eyes (mm)	p	Orientation	Right eyes (mm)	Left eyes (mm)	p
Nasal to temporal	13.64 ± 0.55	13.74 ± 0.40	0.18	11.74 ± 0.42	11.82 ± 0.47	0.25	1.95 ± 0.56	1.92 ± 0.55	0.91	Nasal	2.83 ± 0.32	2.86 ± 0.24	0.48
											1.94 : 3.48	2.11 : 3.53	
	11.69 : 13.99	12.02 : 14.00		10.73 : 12.36	10.35 : 12.36		0.41 : 3.20	0.38 : 3.19		Temporal	2.99 ± 0.31	2.99 ± 0.29	0.99
											2.14 : 3.83	1.95 : 3.56	
										p	< 0.001	< 0.001	NA
Superior to inferior	13.65 ± 0.54	13.75 ± 0.38	0.16	11.52 ± 0.45	11.55 ± 0.46	0.67	2.20 ± 0.57	2.22 ± 0.51	0.30	Superior	3.21 ± 0.31	3.17 ± 0.26	0.35
											2.17 : 3.74	2.32 : 3.89	
	11.76 : 13.99	12.10 : 14.00		10.28 : 12.36	10.34 : 12.36		0.65 : 3.20	0.78 : 3.41		Inferior	3.02 ± 0.48	3.15 ± 0.30	0.03
											1.25 : 3.79	2.19 : 3.67	
p	0.91	0.83	NA	< 0.001	< 0.001	NA	0.02	< 0.001	NA	p	0.61	< 0.001	NA

**Fig 4:**
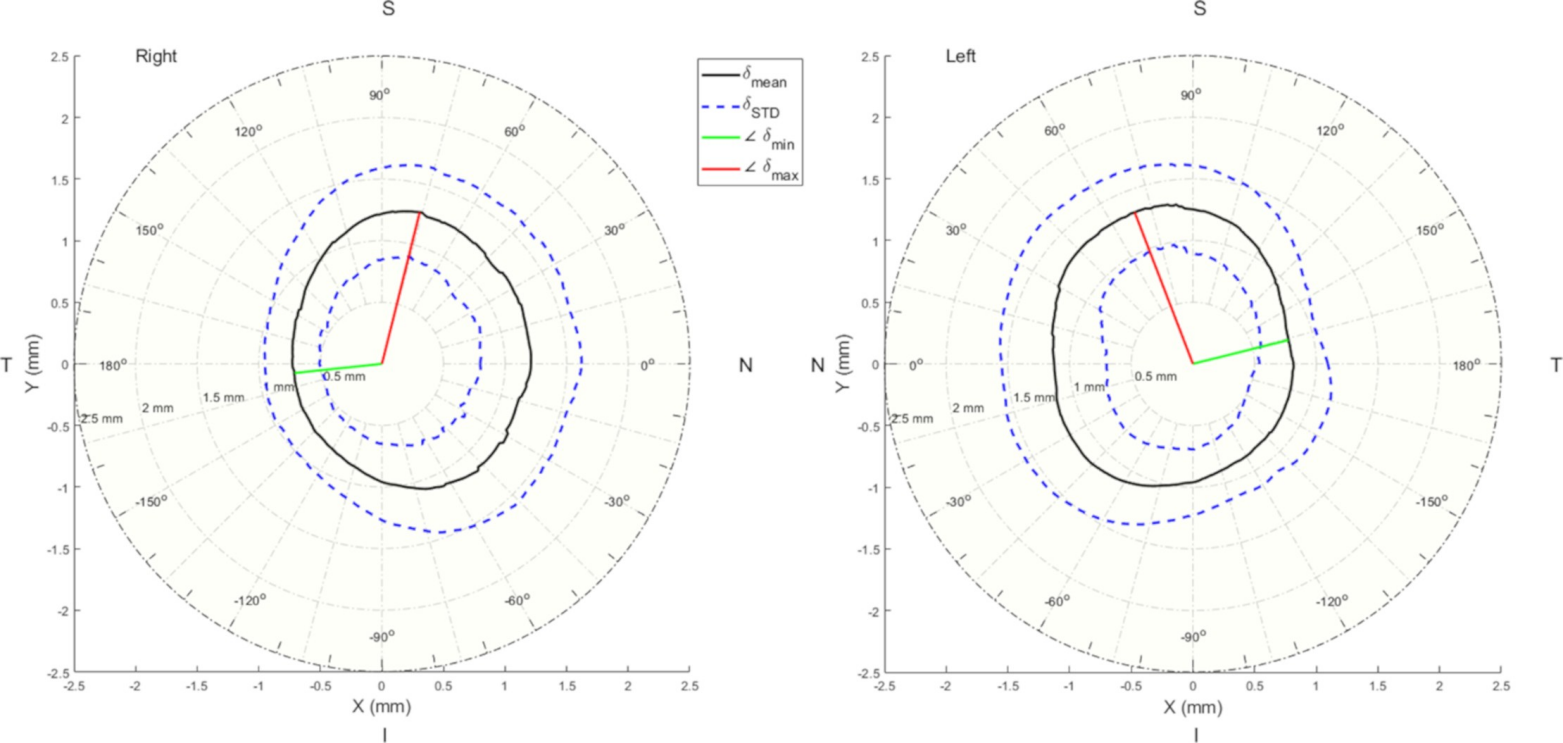
Mean difference (*δ*) between the limbus contours and the white-to-white contours for right and left eyes. N, T, S & I stand for nasal, temporal, superior and inferior sides respectively. The red line is the angle at which the limbus contour recorded the maximum diversion from the white-to-white contour, while the green line is the angle of the minimum diversion.

The difference (δ) between the limbus and the visible iris boundary contours has been determined in all directions, Fig 4. The results showed that the limbus contour was always bigger than the visible iris boundary in all directions, Table 2. In the nasal-temporal direction, the differences were 1.95±0.56 mm for right eyes and 1.92±0.55 mm for left eyes. However, in the superior-inferior direction the differences were 2.2±0.57 mm and 2.22±0.51 mm for right and left eyes, respectively. There were significant differences between the nasal-temporal direction and the superior-inferior direction (p<0.001, p<0.001) and insignificant differences between right and left eyes in both directions (p = 0.91, p = 0.3).

**Fig 5:**
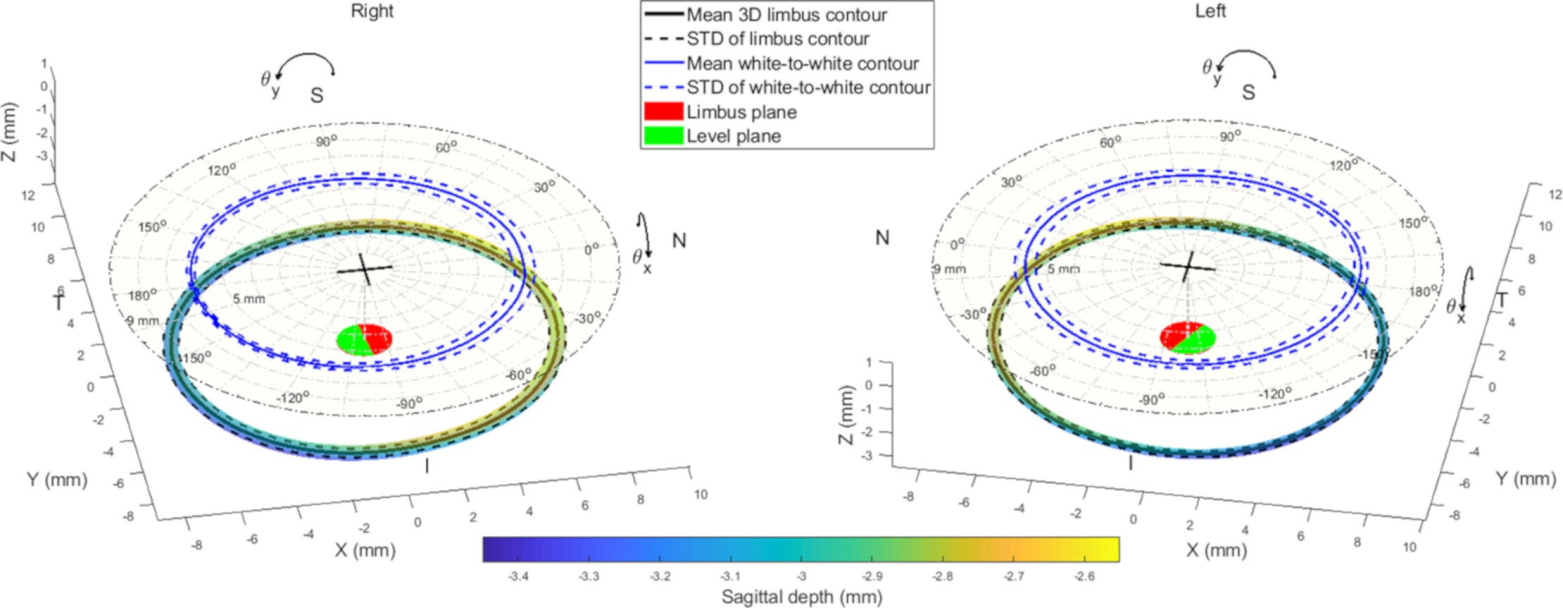
Location of the mean 3D limbus contour (black line) surrounded by standard deviation (STD) as a 3D tube and the mean white-to-white contour for right and left eyes. N, T, S & I stand for nasal, temporal, superior and inferior sides respectively. The red plane is the 3D limbus best fit plane and the green plane is the same plane after being levelled to be normal to the Z-axis. The origin of each plot is marked by a black cross which represent the position of the average corneal apex.

The right eyes’ mean limbus contour tilt around the X-axis θ_*x*_ was found to be -0.3±1.35° with a variation range of -3.51:4.04° measured from the positive Y-axis. However, the right eyes’ mean limbus contour tilt around the Y-axis θ_*y*_ was 1.76±0.9° with a variation range of -0.04°:4.28° measured from the positive X-axis. Likewise, the left eyes’ mean limbus contour tilt around the X-axis θ_*x*_ was 0.77±1.25° with a variation range of -3.02°:3.59° measured from the positive Y-axis and the mean limbus contour left eye tilt around the Y-axis θ_*y*_ was -1.54±0.89° with a variation range of -4.95°:0.04° measured from the positive X-axes, Fig 5. Statistical analysis revealed significant differences among both the same side eye tilt angles around the Cartesian axes (p<0.001) and between the right and left eye tilt angles (p<0.001).

## Discussion

This study aimed to present a novel non-parametric method for detecting the eye limbus in three dimensions and a new dynamic method for measuring the visible iris boundary. While measuring white-to-white distances clinically with a reasonable accuracy is hypothetically possible, locating the limbus positions clinically is a real challenge. The study provides a novel 360° numerical polar map of the radial distance between the limbus contours and the white-to-white contours for both right and left eyes, therefore, by measuring the white-to-white distances in a clinic at a certain angle and then use the map in Fig 4, clinicians should be able to get a good estimate of limbus dimensions. The results of the dynamic method of detecting the white-to-white visible iris contour has revealed that the average nasal-temporal white-to-white distance was found to be 0.26 mm greater than the average superior-inferior white-to-white distance with insignificant differences between bilateral eyes either nasal-temporal (p = 0.25) or superior-inferior (p = 0.67) distances.

With the method proposed in this study, it has been observed that the limbus diameter as a 3D contour is less variable between the nasal-temporal direction and superior-inferior direction with insignificant differences between bilateral eyes (p<0.01). It has also been observed that the limbus contours were tilted and the mean 3D limbus contour was higher on the nasal and inferior sides. However, the 3D limbus contour sagittal depth analysis showed significant differences between right and left eyes in all orientations except on the inferior side, Table 2, the limbus contour tilt angles analysis showed significant tilt between right and left eyes in all orientations. This is due to tilt angles being dependent on the limbus diameter at each orientation and the sagittal depth on both sides of this orientation rather than the single-sided sagittal depth only. The results showed that the 3D limbus contour was always tilted towards the temporal inferior direction, the right eyes were more tilted temporally and left eyes were more tilted inferiorly.

The ESP instrument’s software calculates the limbus location by fitting two spheres to the cornea and the sclera and determining their intersection. Obviously, fitting a sphere to an astigmatic or keratoconic eye is not ideal as the best fit sphere will take an average position between troughs and peaks on the surface of the eye. As a result, this technique may not be reliable for keratoconic or even astigmatic eyes.

The white-to-white distance is used as a reference marker in several clinical applications. Despite having inaccuracies in its correlation to the limbus and the ciliary sulcus, a more precise measurement of its size will reduce the error coming from these approximations [30]. As the limbus is where stem cells resides and about 2.4% to 5% of contact lenses wearers develop signs of limbal stem cell deficiency [31], a proper fitted corneal or scleral contact lens should not sit on the limbus. Hence, in order to fit a contact lens to an eye and achieve the best centration without risking limbal stem cell deficiency, the limbus shape should be well defined. However, the measurement method currently available is the horizontal visible iris diameter, which is used as a surrogate for the limbus size after applying empirical correction factors [32]. Knowing the accurate position of the limbus, and also the analysis of the circumferential variations, will lead to improved contact lens design.

The white-to-white distance has another clinical application as it is used to estimate the iris prosthesis size for patients with aniridia. In this case, the prosthesis size is also calculated by empirically adding a correction a factor to the white-to-white distance. The sizing is important to maintain good centring and avoid excessive contact with peripheral structures which can cause inflammation and glaucoma [33]. Another current clinical use of the white-to-white distance is to estimate the ciliary sulcus size when selecting the diameter of a phakic intraocular lens (pIOL). This type of lens must have its haptics supported in the sulcus and maintain a gentle slope, in order to avoid the contact with the crystalline lens just below and with the iris above it. The correct sizing of the pIOL is related to the sulcus size and is important to avoid clinical complications. A lens that is too short will affect the aqueous flow and the metabolism of the subcapsular epithelial cells of the lens causing a cataract [34]. A lens that is too long will press on the iris root above it, causing sight-threatening complications which could be as serious as angle closure glaucoma [35, 36]. A better determination of the white-to-white distance will be important to reduce these sizing errors and complications for these clinical applications.

The white-to-white distance is used as a predictor of the limbus, sulcus, and effective intraocular lens position (ELP) in some important clinical applications [37]. In the centring process for contact lenses, the horizontal visible iris diameter is used after adding empirical correction factors [32]. Knowing the accurate position of the limbus could improve these processes, and also the analysis of the circumferential variation measurements will lead to improved contact lens design. The same white-to-white diameter, despite being a rough and inaccurate approximation of the limbus and sulcus diameter, is used for selecting the diameter of phakic IOLs and to estimate the ELP in modern formulas [38, 39]. Therefore, having a precise way of measuring white-to-white diameter around 360°, as described in this study, would reduce the inaccuracies associated with this estimation. Also, the true limbus dimension is a better surrogate than the white-to-white diameter.

## Supporting information

S1 Table Limbus data. (XLSX)
